# Increased Osteoclastogenesis in Absence of TG2 Is Reversed by Transglutaminase Inhibition—Evidence for the Role for TG1 in Osteoclast Formation

**DOI:** 10.3390/cells12172139

**Published:** 2023-08-24

**Authors:** Sahar Ebrahimi Samani, Mari T. Kaartinen

**Affiliations:** 1 Faculty of Medicine and Health Sciences (Division of Experimental Medicine), McGill University, Montreal, QC H3A 0C7, Canada; 2 Faculty of Dental Medicine and Oral Health Sciences (Biomedical Sciences), McGill University, Montreal, QC H3A 0C7, Canada

**Keywords:** transglutaminases, TG2, TG1, Factor XIII-A, osteoclastogenesis

## Abstract

Osteoclasts are multinucleated, bone-resorbing giant cells derived from monocyte–macrophage cell lines. Increased bone resorption results in loss of bone mass and osteoporosis. Osteoclast and bone marrow macrophages have been shown to express three TG enzymes (TG2, Factor XIII-A, and TG1) and TG activity to regulate osteoclast differentiation from bone marrow macrophages in vitro. In vivo and in vitro studies have demonstrated that the deletion of TG2 causes increased osteoclastogenesis and a significant loss of bone mass in mice (*Tgm2*−/− mice). Here, we confirm that TG2 deficiency results in increased osteoclastogenesis in vitro and show that this increase can be reversed by a TG inhibitor, NC9, suggesting that other TGs are responsible for driving osteoclastogenesis in the absence of TG2. An assessment of total TG activity with 5-(biotinamido)-pentylamine, as well as TG1 and FXIII-A activities using TG-specific Hitomi peptides (bK5 and bF11) in *Tgm2*−/− bone marrow flushes, bone marrow macrophages, and osteoclasts, showed a significant increase in total TG activity and TG1 activity. Factor XIII-A activity was unchanged. Aspartate proteases, such as cathepsins, are involved in the degradation of organic bone matrix and can be produced by osteoclasts. Moreover, Cathepsin D was shown in previous work to be increased in TG2-null cells and is known to activate TG1. We show that Pepstatin A, an aspartate protease inhibitor, blocks osteoclastogenesis in wild-type and Tgm2−/− cells and decreases TG1 activity in Tgm2−/− osteoclasts. Cathepsin D protein levels were unaltered in Tgm2−/−cells and its activity moderately but significantly increased. *Tgm2*−/− and *Tgm2*+/+ bone marrow macrophages and osteoclasts also expressed Cathepsin E, and Renin of the aspartate protease family, suggesting their potential involvement in this process. Our study brings further support to the observation that TGs are significant regulators of osteoclastogenesis and that the absence of TG2 can cause increased activity of other TGs, such as TG1.

## 1. Introduction

Bone tissue is renewed throughout life to maintain appropriate bone strength and mass in a process referred to as bone remodeling [1]. This renewal process involves the removal of old bone by bone-resorbing cells, osteoclasts, and their replacement with new bone by bone-forming cells, osteoblasts [2]. Cellular defects in bone remodeling and imbalanced coupling between the bone cells lead to osteoporosis [3], a skeletal disease characterized by decreased bone mass and increased prevalence of bone fractures [4]. Osteoclastogenesis involves two main stages, recruitment and the differentiation of monocyte–macrophage lineage cells into mononuclear pre-osteoclasts, followed by their fusion to multinucleated osteoclasts [5]. Osteoclastogenesis is promoted by monocyte–macrophage colony-stimulating factor (M-CSF) and receptor activation of NF-кB ligand (RANKL) [5,6]. M-CSF mediates the proliferation and sustains the survival of osteoclast precursors [7]. RANKL mediates osteoclast differentiation and survival by binding to its receptor, RANK [8]. The ligation of RANK by its ligand leads to the activation of a wide array of downstream signaling, resulting in the differentiation of pre-osteoclasts to osteoclasts that express a dendritic cell-specific transmembrane protein (DC-STAMP) and osteoclast stimulatory transmembrane protein (OC-STAMP), which allow for fusion of the cells [9,10,11,12]. Osteoclasts also express tartrate-resistant acid phosphatase (TRAP), matrix metalloproteinase 9 (MMP9), and Cathepsin K (CTSK), which are secreted into the acidic resorption pit to digest the organic extracellular matrix (ECM) of bone [13]. The expression and activity of proton pumps in the ruffled border of the cell in the sealing zone (such as V-ATPase 3) create a highly acidic environment that dissolves minerals prior to ECM digestion [14].

The transglutaminase (protein glutamine-glutamyltransferase, TGase, TG, EC 2.3.2.13) family consists of nine structurally and functionally related enzymes. Of the nine TGs, erythrocyte membrane protein band 4.2 has lost its enzymatic activity during evolution and acts as a structural protein. TG1-7 and Factor XIII-A (FXIII-A) are active enzymes that can form isopeptide bonds/crosslinks between the carboxyamide of glutamine (Q) and the amine group of lysine (K) residues in a Ca^2+^-ion- and thiol-dependent way, which may lead to structural changes in proteins and their network assembly, which, in turn, can cause different cellular responses [15,16]. Our work demonstrates the presence of three transglutaminases, TG1, TG2, and FXIII-A, in macrophage lineage cells and osteoclasts, and we showed that the inhibition of TG activity with three different TG inhibitors, NC9, T101, and Z006, results in the complete blockage of osteoclastogenesis [17]. NC9 also blocks resorption activity [17]. These data introduce TGs as important regulators of osteoclast differentiation, fusion, and migration [17]. Kim et al. demonstrated in vitro and in vivo that TG2 deletion (*Tgm2*−/− mice) resulted in a dramatic decrease in trabecular bone mass and increased osteoclastogenesis, suggesting that TG2 is an inhibitor of osteoclastogenesis [18]. The authors substantiated this finding by also showing that TG2 overexpression suppresses RANKL-induced osteoclast differentiation, via mechanisms involving NF-κB- and Blimp1-dependent pathways and the downregulation of anti-osteoclastogenic factors like IRF8 and MafB [18]. The skeletal phenotyping of *Tgm2*−/−; *F13a1*−/− mice generated by us also revealed a dramatic loss of bone mass and an increase in osteoclastogenesis; however, the increased osteoclastogenesis of *Tgm2*−/−; *F13a1*−/− monocytes was completely blocked by NC9, a transglutaminase inhibitor [19]. This finding suggests that the remaining TG, i.e., TG1, could be driving osteoclastogenesis in the absence of TG2 and FXIII-A. However, the exact regulatory pathway of transglutaminases during osteoclastogenesis has not being investigated. To gain a better understanding of the circuitry role of TGs, we explored whether the observed increase in osteoclastogenesis in *Tgm2*−/− cells involves the activity of the other TGs in monocytes/macrophages and osteoclasts. Understanding the molecular mechanisms of osteoclastogenesis will allow for the development of novel strategies to target bone resorption and combat the loss of bone mass in osteoporosis. The knowledge arising from this current work brings further support to the potential of TGs, specifically TG1, as targets to limit bone resorption, which will, in turn, facilitate the design of more specific TG inhibitors that could restore balance in bone remodeling.

## 2. Materials and Methods

### 2.1. Animals

The generation of *Tgm2* knockout mice has been previously described [20]. Animals were housed in a pathogen-free environment and maintained under standard conditions. All animal experiments were performed according to the Guidelines for Animal Experimentation and were approved by the Animal Care Committee of McGill University. Mice aged 6 to 10 weeks old (TG2 KO) with age-matched control groups were used in this study to isolate bone marrow and bone marrow macrophages. Genomic DNA was extracted from tail biopsies using the Mouse Direct PCR Kit (Bimake, Houston, TX, USA). Wild-type (WT) mice for breeding were purchased from Jackson Laboratories.

### 2.2. Reagents and Antibodies

MEM Alpha (αMEM) (12561-056), penicillin-streptomycin, L-glutamine, and sodium pyruvate were purchased from Gibco (Burlington, ON, Canada). Fetal bovine serum was obtained from Hyclone (Waltham, MA, USA). Human M-CSF and human sRANK ligand were obtained from PeproTech (Rocky Hill, NJ, USA). 5-(Biotinamido)pentylamine (bPA) was obtained from Thermo Fisher Scientific (Rockford, IL, USA). Biotinylated “Hitomi-substrate peptides” bK5 (Biotinyl-YEQHKLPSSWPF) and bF11 (Biotinyl-DQMMLPWPAVAL), and their control peptides, bK5QN (Biotinyl-YENHKLPSSWPF) and bF11QN (Biotinyl-DNMMLPWPAVAL), were synthesized by Biomatik Corporation (Cambridge, MA, USA). All other reagents were purchased from Sigma-Aldrich (Oakville, ON, Canada) or Fisher Scientific (Hampton, NH, USA). Tartrate-resistant acid phosphatase (TRAP) Staining Kit was purchased from Cosmo Bio (Tokyo, Japan). Cathepsin D Activity Assay Kit (Fluorometric) (ab65302) and Mouse Cathepsin D ELISA kit (ab239420) were purchased from Abcam (Cambridge, UK). Pepstatin A (77170) was purchased from Sigma. Mouse monoclonal antibody against biotin (BN-34) and rabbit polyclonal actin antibody (SAB4301137) were obtained from Sigma. Polyclonal antibody to mouse Cathepsin D was obtained from R&D Systems (AF1029). Rabbit polyclonal TG1 antibody (12912-3-AP) was obtained from Proteintech. The secondary antibody HRP-linked anti-mouse IgG was obtained from GE Healthcare (Mississauga, ON, Canada). HRP-linked anti-rabbit IgG was purchased from Cell Signaling (Whitby, ON, Canada).

### 2.3. Bone Marrow Macrophage Isolation and Osteoclast Cultures

A medium composed of αMEM as the base, 1% penicillin–streptomycin, 1% L-glutamine solution, 1% sodium pyruvate, and 10% FBS as supplements was used in this study. Murine bone marrow cells were extracted from 6- to 10-week-old *Tgm2* knockout mice and littermate wild-type mice cultured overnight with 25 ng/mL M-CSF. After 24 h, non-adherent cells were collected, and then, plated at 5 × 10^4^ cells/cm^2^ for another 48 h in the presence of M-CSF. The adherent cells were M-CSF-dependent bone marrow-derived macrophages (BMMs) and were used as osteoclast precursors. Osteoclast differentiation was induced by treating cells with 50 ng/mL M-CSF and 50 ng/mL RANKL for 6 days with two medium changes on day 2 and day 4. The BMMs treated with only M-CSF were considered a negative control. The experimental treatments included a TG inhibitor, NC9 (40 µM), and a CTSD inhibitor, Pepstatin A (80 µM), for 6 days.

### 2.4. TRAP Staining and Activity Assay

On day 6 of culture, cells were fixed with 3.7% formaldehyde, and stained with a TRAP Staining Kit following the manufacturer’s protocol. TRAP-positive (TRAP+) multinucleated cells consisting of 3 nuclei or more were considered matured osteoclasts. After staining, cells were washed with dH_2_O, and images were taken using a Zeiss Axioscope 5 microscope. A TRAP activity assay was performed using a TRAP staining kit according to the manufacturer’s recommendations on culture supernatant; the supernatant cultures were incubated with reaction buffers for 3 h in the dark at 37 °C, and then, read at 540 nm using a microplate reader (TECAN infinite F200 PRO, Männedorf, Switzerland).

### 2.5. Protein Extraction and Western Blotting

Cells at their experimental endpoints were washed with PBS and incubated with ice-cold cell lysis buffer containing 50 mM Tris (pH 7.5), 0.5 M NaCl, 2% Igepal, 1% protease inhibitor cocktail, and 1% phosphatase inhibitor cocktail for 30 min on ice. The lysate was prepared via scraping and sonication for 15 s followed by centrifugation at 14,000× *g* for 15 min at 4 °C. Protein concentration was measured using the BCA Protein Assay Kit (Thermo Fisher Scientific). For Western blotting, 30 µg of proteins were loaded onto 10% SDS-polyacrylamide gels in denaturing conditions and were subsequently transferred to a PVDF membrane (Bio-Rad, Mississauga, ON, Canada). After blocking with 5% nonfat skim milk in Tris-buffered saline with 0.1% Tween 20 (TBST), the membrane was incubated with the primary antibody overnight at 4 °C. This was followed by a 1 h incubation with secondary antibodies conjugated with HRP. Immunoreactivity was detected using an ECL Plus kit (GE Healthcare) and visualized using the ChemiDoc™ Touch Imaging System. Western blot band intensities of each lane were quantified using the NIH Image J (v. 1.41) program.

### 2.6. In Vitro TG Activity Assay

The in vitro TG activity assay was performed as follows: The proteins of each sample were extracted as above. A total of 20 μg of protein was incubated with either 5-(biotinamido)pentylamine (bPA) (500 μM) or biotinylated “Hitomi peptides”, bF11 (40 μM), and bK5 (150 μM) and their control peptides, where TG-reactive glutamine-residue (Q) was changed into asparagine (N). Incubations were performed in reaction buffer containing 1 mM dithiothreitol (DTT) and 3 mM CaCl_2_ 10 mM Tris-HCl (pH 8.0) for 2 h at 37 °C. Reaction products were loaded on an SDS–PAGE and analyzed via Western blotting as above, using an anti-biotin antibody to visualize the covalent incorporation of bPA or biotinylated peptides to their substrates as an indication of TG activity in the extracts [17]. Quantification was carried out from two separate cell culture extracts in triplicate. Supplemental Figure S1 shows no biotin-detection in negative controls. Band intensities were quantified as above.

### 2.7. RNA Extraction and RT-qPCR

RNA was extracted using the RNeasy Mini Kit (Qiagen, Venlo, The Netherlands). After measuring the concentration of RNA using a spectrophotometer, cDNA was synthesized from 1 μg total RNA using the High-Capacity cDNA Reverse Transcription Kit (Applied Biosystems, Foster City, CA, USA). Subsequently, qPCR was performed using the StepOnePlus Real-Time PCR System (Applied Biosystems) in a 20 µL reaction volume consisting of the following: 9 µL (50 ng) synthesized cDNA, 10 µL TaqMan Fast Advanced Master Mix, and 1 μL of each TaqMan Gene Expression Assay. TaqMan^®^ Fast Advanced Master Mix and primers were purchased from Applied Biosystems. The expression level of *Tgm1* (Mm00498375_m1), *F13a1* (Mm00472334_m1), and markers of osteoclast differentiation or fusion, including *Ctsk* (Mm00484039_m1), *Dcstamp* (Mm04209234_m1), *Mmp9* (Mm00442991_m1), *Ctsd* (Mm00515587_m1), *Ctse* (Mm00456010_m1), and Ren1 (Mm02342887_mH), were assessed and normalized to Gapdh (Mm99999915_g1).

### 2.8. Cathepsin D Protein and Activity Assays

Cathepsin D activity was measured using a kit from Abcam (ab65302, Cambridge, UK) according to the manufacturer’s instructions. Cells at their experimental endpoints were washed twice with cold PBS, digested with 0.25% trypsin, and resuspended in PBS. Next, the cells were collected via centrifugation at 1000 rpm at 4 °C and resuspended in cell lysis buffer on ice for 10 min. The supernatants were obtained via centrifugation at 16,000× *g* for 5 min at 4 °C, followed by mixing the cell lysis samples with a reaction buffer and incubating them with the substrate at 37 °C for 2 h. Finally, the absorbance of the solution was quantified using a fluorometer at Ex/Em = 328/460 nm. Cathepsin D protein levels were determined using a commercial Enzyme-Linked Immunosorbent Assay-ELISA kit from Abcam, which is a colorimetric-based assay. After following the manufacturer’s instructions, the OD of each sample at 450 nm was recorded using a microplate reader, and the Cathepsin D level in each sample was determined.

### 2.9. Statistical Analysis

Data were analyzed using GraphPad Prism software (version 9). Results are expressed as the mean ± SEM (standard error of the mean). N-value represents three mice (*n* = 3; both +/+ and −/−). Each experiment was performed in triplicate. Data were analyzed via one-way and two-way analysis of variance (ANOVA), followed by a multiple-comparison Tukey post hoc test. When comparing two groups, Student’s *t*-test was used. The differences were considered statistically significant for *p*-values < 0.05 (* *p* < 0.05, ** *p* < 0.01, *** *p* < 0.001, **** *p* < 0.0001, ns: not significant).

## 3. Results

### 3.1. Increased Osteoclastogenesis in the Absence of TG2

**Figure 1:**
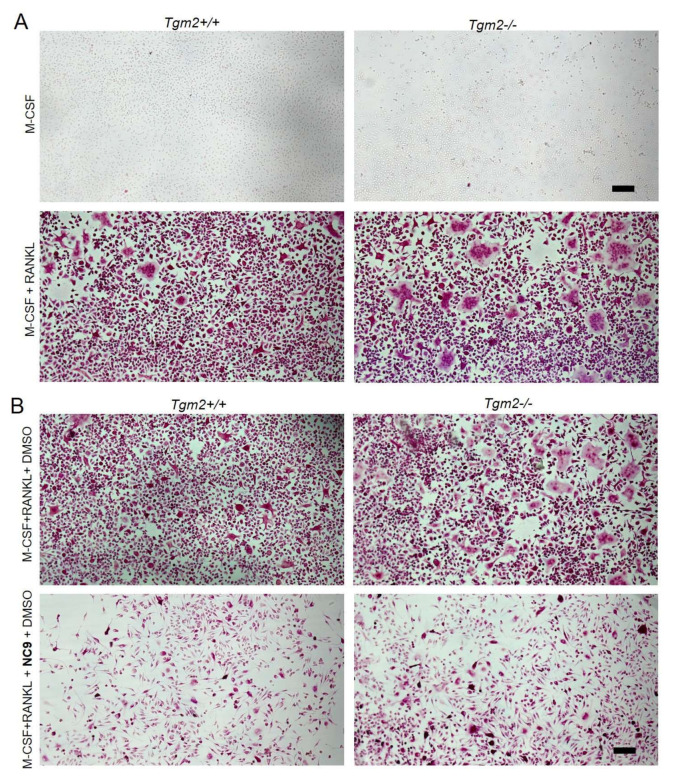
Increased osteoclastogenesis of *Tgm2*−/− BMMs and its reversal by TG inhibitor NC9 visualized via TRAP staining. Bone marrow macrophages (BMMs) of *Tgm2*−/− were cultured with M-CSF and RANKL in the presence and absence of TG inhibitor NC9 for 6 days and compared to the wild-type group. Cells were stained for tartrate-resistant acid phosphatase (TRAP) at the end point. (**A**) *Tgm2*−/− BMM resulted in more and larger osteoclasts compared to controls. (**B**) NC9 treatment effectively reversed the increased osteoclastogenesis of *Tgm2*−/− BMMs cells. DMSO was used as a vehicle for NC9 and an experiment with M-CSF+RANKL+DMSO is presented as a control culture. The images are representative of three separate experiments (*n* = 3).

**Figure 2:**
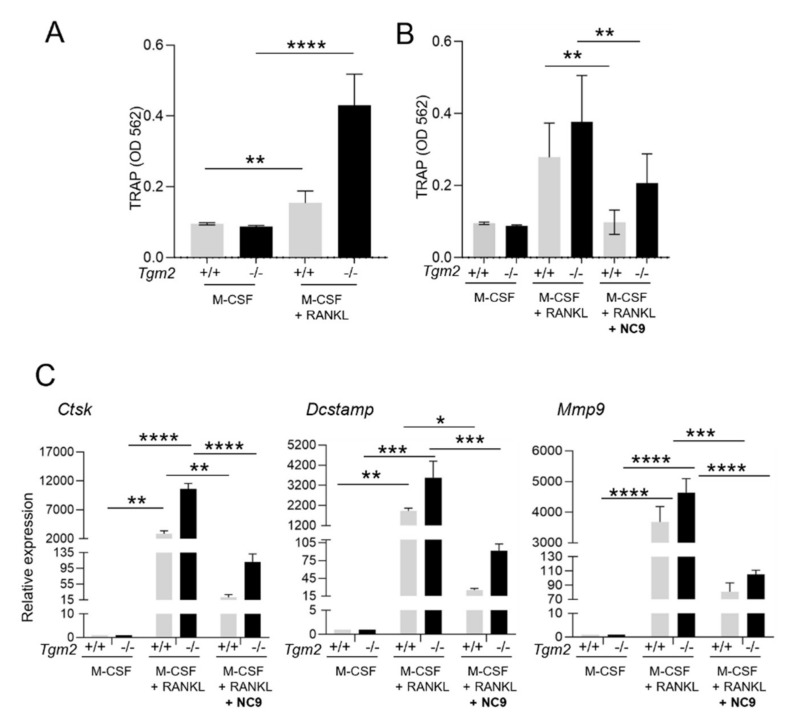
Quantification of the increased osteoclastogenesis of *Tgm2*−/− BMMs via TRAP quantification and RT-qPCR analysis. Bone marrow macrophages (BMMs) from *Tgm2*−/− were cultured with M-CSF and RANKL, with and without NC9, for a period of 6 days, and were then compared to the wild-type group. (**A**) Quantification of TRAP activity in the culture supernatant showed a significant increase in the osteoclastogenesis of *Tgm2*−/− cells. (**B**) NC9 reversed the increase in TRAP activity significantly. (**C**) The mRNA levels of the osteoclast marker genes *Ctsk*, *Dcstamp*, and *Mmp9* were measured via RT-qPCR. The result confirmed the increase in *Tgm2*−/− cells and its reversal by NC9. The data are expressed as mean ± SEM, *n* = 9. * *p* < 0.05, ** *p* < 0.001, *** *p* < 0.001, **** *p* < 0.0001.

To confirm the increased osteoclastogenesis in the absence of TG2, bone marrow macrophages (BMMs) were isolated from *Tgm2*−/− and the littermate controls (*Tgm2*+/+) and osteoclastogenesis was induced with M-CSF and RANKL. The potential role of TG activity in the increased osteoclastogenesis of *Tgm2*−/− osteoclasts was also investigated by adding a TG inhibitor, NC9, to the cultures during differentiation. Staining for tartrate-resistant acid phosphatase (TRAP) as a marker for osteoclasts confirmed the previous findings that the absence of TG2 does significantly promote osteoclast formation in cultures. A visible increase in TRAP-positive (TRAP+) mononuclear cells and large osteoclasts was observed (Figure 1). Counting the number of TRAP+ osteoclasts (nuclei ≥ 3) confirmed a significant increase in *Tgm2*−/− cultures (Supplemental Figure S2). Similarly, an increase in osteoclasts was visible via actin-staining of the cells, showing an increase in large, podosome-structure-containing cells (Supplemental Figure S3). The formation of TRAP+ osteoclasts was visibly reduced by NC9 (Figure 1). The TRAP level in the osteoclast culture supernatant was increased in *Tgm2*−/− osteoclasts, and this was inhibited by NC9 (Figure 2A,B). Increased osteoclastogenesis in *Tgm2*−/− and its reversal by NC9 were further confirmed via RT-qPCR analysis of the osteoclast markers *Mmp9*, *Ctsk*, and *Dcstamp* (Figure 2C). This strongly suggests that another TG or other TGs are responsible for driving osteoclastogenesis in *Tgm2*−/− cells.

### 3.2. Increased Total TG Activity and TG1 Activity in Osteoclasts in the Absence of Tgm2

**Figure 3:**
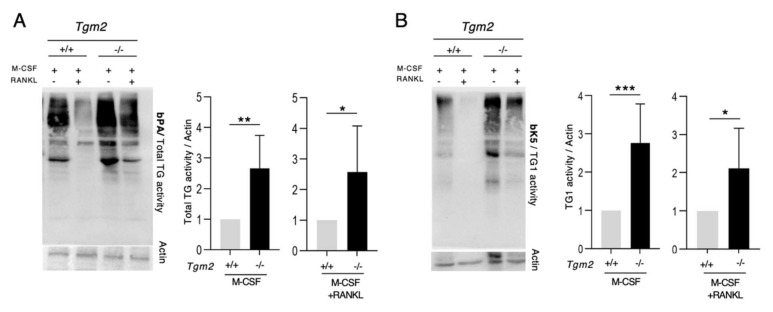
*Tgm2*−/− bone marrow macrophages and osteoclasts have increased total TG and TG1 activities. Bone marrow macrophages (BMMs) isolated from *Tgm2*−/− and *Tgm2*+/+ mice were treated with M-CSF to maintain cells as BMMs or M-CSF+RANKL to induce osteoclastogenesis. On day 6, cells were extracted and TG activities were assessed with 5-(biotinamido)pentylamine and Hitomi peptides bK5 and bF11, which assess total activity, TG1 activity, and FXIII-A activity, respectively. Western blotting detection for biotin to visualize biotin incorporation with substrates and the quantification of band intensities show the following: (**A**) significantly higher total TG activity in *Tgm2*−/− BMMs and *Tgm2*−/− osteoclasts compared to control cells, and (**B**) significantly higher TG1 activity in *Tgm2*−/− BMMs and *Tgm2*−/− osteoclasts compared to control cells. Western blot experiments were normalized with respect to β-actin using NIH Image J. All statistical analyses were performed using Student’s T-test between knockout cells and WT cells (set to 1). Graphs do not reflect changes in activity between BMMs and osteoclasts. The data are expressed as the mean ± SEM, *n* = 6. * *p* < 0.05, ** *p* < 0.001, *** *p* < 0.001.

Our previous work suggested the involvement of FXIII-A and TG1 in the differentiation and fusion stages of osteoclastogenesis, making them plausible contributors the TG activity in *Tgm2−/−* osteoclasts. Hitomi peptides have been used to measure the activity of specific TGs in a system where multiple TGs are present [21,22,23]. These peptides were developed by the research group of Dr. Kiyotaka Hitomi using phage display screening of the preferred glutamine (Q) containing peptide sequences for each TG [21,22,23]. This elegant development scenario identified F11 and K5 for the detection of the activities of FXIII-A and TG1, respectively [21,22,23]. The Q-residue crosslinks to the K-residues of substrate proteins, which can be used for the in situ visualization of TG activity as well as detection, and the purification of K-containing substrates. Their control peptides, Q-residues, are replaced with N-residues, which makes them inactive towards TGs [21,22,23]. In this study, biotinylated versions of the peptides F11 and K5 were used here, which allowed for their easy detection in substrate proteins. In addition, the classical primary amine substrate 5-(biotinamido)pentylamine, bPA, was used as a general activity ‘probe’ to evaluate total TG activity. bPA incorporates with the Q-residues of substrate proteins and acts as a substrate for all TGs. Validation of the use of peptides and their controls is presented in Supplemental Figure S1. The analysis of total TG activity with bPA during osteoclastogenesis showed significantly higher activity in *Tgm2*−/− in both BMMs (M-CSF-treated) and osteoclasts (M-CSF+RANKL-treated) (Figure 3A). In addition, activity assays using the bK5 peptide showed a significant increase in TG1 activity in knockout BMMs and osteoclasts (*Tgm2*−/−) compared to the WT controls (*Tgm2*+/+) (Figure 3B). FXIII-A activity was slightly but significantly increased in *Tgm2*−/− BMMs compared to WT, but no change was seen in osteoclasts, which have very low or no FXIII-A activity (Supplemental Figure S4). Quantification was performed between the WT and knockout cells, i.e., the WT values were set to 1. Thus, the graphs do not represent decreases or increases between BMMs and osteoclasts; however, altered activities are clearly seen in the WBs. The TG activities in *Tgm2*−/− bone marrow flushes were also examined, and as seen in Supplemental Figure S5, bone marrow total TG activity was dramatically higher in the absence of TG2. Furthermore, TG1 activity showed an increase, whereas FXIII-A activity did not show an increase, in the bone marrow flushes. All activities were quantified using Image J and normalized to β-actin, which has been used for the normalization of TG activity in the WB method in numerous studies [24,25,26,27,28,29]. Our examination of the mRNA expression levels of *Tgm1* and *F13a1* in BMMs and osteoclasts showed no significant differences (Supplemental Figure S6), suggesting that TG2 regulates TG1 protein production or activity.

**Figure 4:**
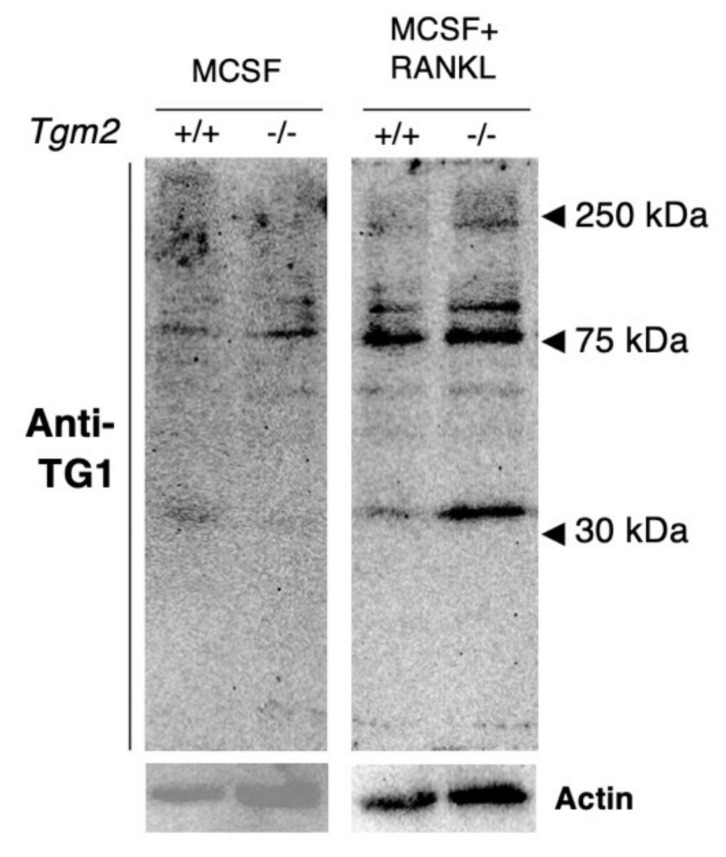
The expression of TG1 protein in BMMs and osteoclasts. The total extracted protein lysates from BMMs and osteoclasts (30 µg) were analyzed via immunoblotting using a specific polyclonal antibody for TG1. The detection of TG1 in osteoclasts revealed multiple molecular weights, indicating the presence of both full-length and fragments of TG1. Fragment of above 30 kDa and high-molecular-weight forms of around 250 kDa appear more prominent in *Tgm2*−/− osteoclast lysates.

The analysis of TG1 protein in BMMs and osteoclasts using the TG1 antibody (12912-3-AP) showed that TG1 protein is, indeed, produced by both cell types (Figure 4). Full-length TG1 is approximately 106 kDa and its proteolytic activation in the skin produces fragments of 100 kDa, 67 kDa, and 33 kDa [30,31]. Interestingly, here, TG1 was found in multiple molecular weights in both +/+ and −/− cells, suggesting its proteolytic activation; however, the TG1 fragment around 30 kDa and the high-molecular-weight form of TG1 were visibly increased in *Tgm2*−/− osteoclasts. The anti-TG1 antibody is developed against a large C-terminal fragment of the enzyme, which allows for the detection of the full length (106 kDa) and the active (cleaved) forms of TG1 (activation occurs in the N-terminus).

### 3.3. Inhibition of Increased Osteoclastogenesis and TG1 Activity in Tgm2−/− Osteoclasts by Aspartate Protease Inhibitor Pepstatin A

We next asked how the absence of TG2 could result in increased TG1 activity in osteoclasts and bone marrow. The appearance of fragments of TG1 suggests that the proteolytic activation of TG1 may be increased in *Tgm2*−/− cells. Several studies have shown the involvement of aspartate protease in the cleavage of organic constituents of the bone matrix [32]. It has also been demonstrated that TG1 can be activated via Cathepsin D-mediated proteolytic cleavage [33]. Interestingly, TG2 was shown to increase Cathepsin D (CTSD) activity as part of the apoptotic process and mediate the balance between cell survival and cell death in mouse embryonic fibroblasts [34]. Therefore, we asked if aspartate proteases are involved in the increased osteoclastogenesis in *Tgm2*−/− cells.

**Figure 5:**
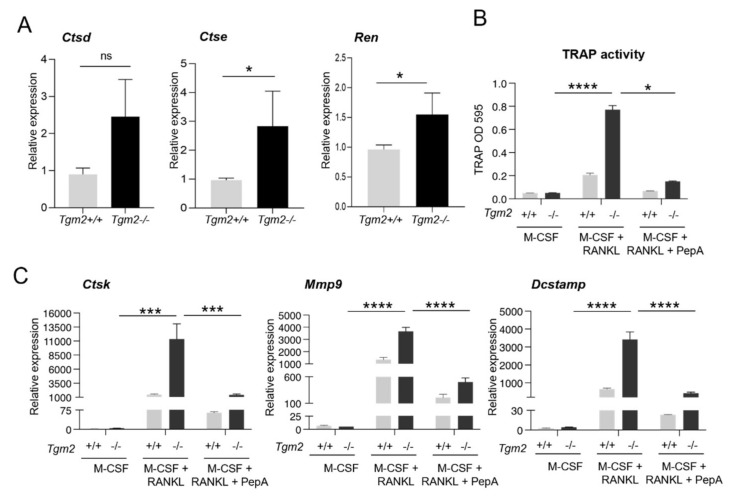
Aspartate protease inhibitor Pepstatin A blocks osteoclastogenesis of WT and *Tgm2*−/− cells. The presence of aspartic protease in *Tgm2*+/+ and *Tgm2*−/− BMMs was measured via RT-qPCR. (**A**) Both WT and knockout BMMs express mRNA of three aspartic proteases, Cathepsin D (*Ctsd*), Cathepsin E (*Ctse*), and Renin (*Ren*), which were all elevated in *Tgm2*−/− BMMs; *Ctse* and *Ren* were found to be significantly higher. (**B**) Quantification of TRAP levels in wild-type and *Tgm2*−/− osteoclasts with and without Pepstatin A (PepA) treatment showed significantly decreased osteoclastogenesis in the PepA treatment group. (**C**) RT-qPCR analysis of osteoclast markers confirmed the effects of PepA: a decrease in the mRNA expression of *Ctsk, Mmp9,* and *Dcstamp* in *Tgm2*−/− osteoclasts and in WT cells. The data are expressed as the mean ± SEM, *n* = 9. * *p* < 0.05, *** *p* < 0.001, **** *p* < 0.0001. ns, not significant.

First, the expression of the aspartic protease family, including *Ctsd* (Cathepsin D), *Ctse* (Cathepsin E), and *Ren* (Renin), was confirmed in BMMs of *Tgm2*−/− and *Tgm2*+/+, and it was observed that they were all elevated, *Ctse* and *Ren* significantly in the absence of TG2 (Figure 5A). Next, the possible involvement of aspartate protease activity in the osteoclastogenesis of *Tgm2*−/− was examined by treating cells with Pepstatin A, an inhibitor of the aspartic protease family. Measurements of TRAP activity in supernatant cultures treated with M-CSF, RANKL, and Pepstatin A on day 6 showed significant inhibition of osteoclast differentiation in both WT and knockout cells (Figure 5B). This was further supported by a significant decrease in the mRNA levels of the osteoclast differentiation and fusion markers *Ctsk*, *Mmp9,* and *Dcstamp* by Pepstatin A (Figure 5C).

**Figure 6:**
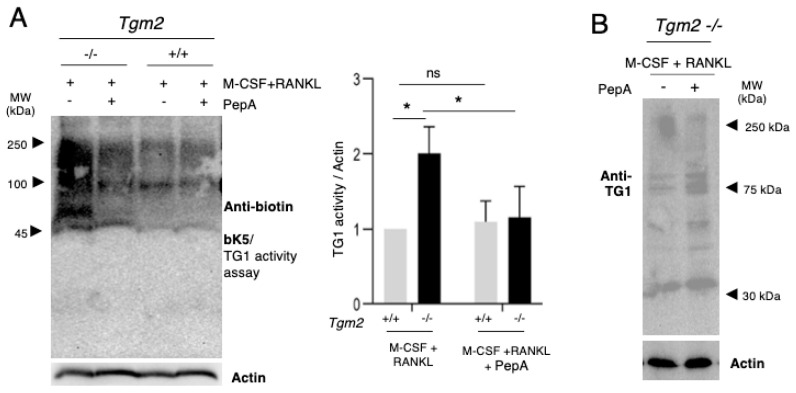
Aspartate protease inhibitor Pepstatin A blocks the increased TG1 activity and increases the presence of full-length TG1 in *Tgm2*−/− cells. (**A**) *Tgm2*+/+ and *Tgm2*−/− BMMs were treated with M-CSF + RANKL for six days in the presence and absence of Pepstatin A (PepA). TG1 activity was assessed using biotin-Hitomi peptide, bK5, via Western blotting method. Biotin incorporation with the substrate proteins in the extracts was detected and band intensities were quantified and normalized to β-actin using NIH Image J. Quantification of the blots shows that PepA decreases TG1 activity significantly in *Tgm2*−/− osteoclasts, suggesting the possible activation of TG1 by an aspartate protease in the mixture, specifically in the absence of TG2. (**B**) Detection of TG1 protein via Western blotting in PepA-treated *Tgm2*−/− osteoclast shows increased full-length TG1 compared to osteoclast cells without Pepstatin A treatment. The data are expressed as the mean ± SEM, *n* = 9. * *p* < 0.05. ns, not significant.

The assessment of TG1 activity in osteoclasts that were grown in the presence or absence of Pepstatin A showed, again, that TG1 activity was significantly increased in *Tgm2*−/− osteoclasts and significantly decreased back to its wild-type activity level with Pepstatin A treatment (Figure 6A). Moreover, Pepstatin A treatment clearly increased the presence of full-length TG1 in the extracts, suggesting that activation may not be occurring (Figure 6B). This strongly suggests that the increase in TG1 activity is mediated by aspartic proteases, specifically in the absence of TG2.

**Figure 7:**
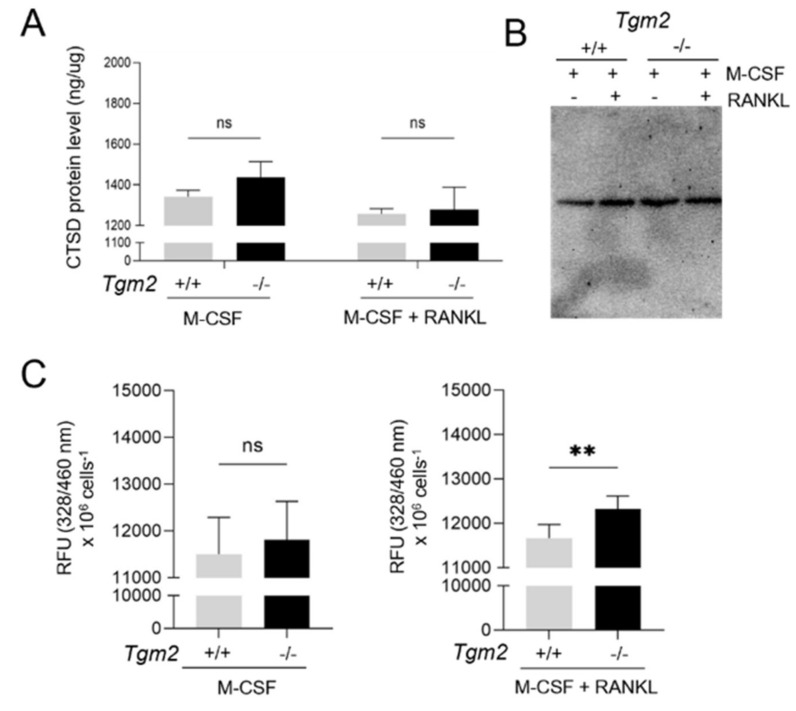
**Cathepsin D activity is increased in *Tgm2***−/− **cells.** Cathepsin D protein levels and activity were measured in BMMs and osteoclasts using a commercial ELISA, Western blotting, and a fluorometric activity kit, respectively. (**A**,**B**) Cathepsin D protein levels were not significantly elevated in *Tgm2*−/− compared to the control group. (**C**) Cathepsin D activity showed a modest but significant increase in *Tgm2*−/− osteoclasts compared to the WT cells. No significant increase was seen in BMMs. The data are expressed as the mean ± SEM, *n* = 9. ** *p* < 0.01. ns, not significant.

Finally, because of the published link between TG2 and Cathepsin D and the activation of TG1 by Cathepsin D, an investigation was conducted to examine whether Cathepsin D activity and protein levels are increased in *Tgm2*−/− osteoclasts. While the protein levels remained the same in the WT and knockout cells in both BMMs and osteoclasts (as analyzed using the ELISA kit and WB) (Figure 7A,B), the Cathepsin D activity (as measured using a fluorometric kit) levels showed a modest but significant increase in *Tgm2*−/− vs. WT osteoclasts (Figure 7C). This suggests that Cathepsin D may be an activator of TG1 in BMMs and in the bone marrow.

## 4. Discussion

Studies from other authors and ourselves have suggested the involvement of three transglutaminases, TG1, TG2, and FXIII-A, in bone resorption and osteoclastogenesis [18,19,35]. Our work on *Tgm2*−/−; *F13a1*−/− double-knockout mice and work from Kim et al. [18] on *Tgm2*−/− mice show dramatic bone loss and increased osteoclastogenesis, demonstrating that TG2 and FXIII-A are inhibitors of osteoclast differentiation. Our observation that the chemical inhibition of TG activity in *Tgm2*−/−; *F13a1*−/−BMMs blocks osteoclastogenesis raises the intriguing possibility that TG1, which is expressed in these double-knockout cells, might be a promoter of osteoclast differentiation. In the present paper, we have examined this circuitry further and confirmed that *Tgm2*−/− BMMs are more ‘potent’ in forming osteoclasts and show increased TG1 activity. The increased osteoclastogenesis of *Tgm2*−/− cells can be inhibited by NC9, suggesting that, indeed, TG1 may be driving the increased osteoclast differentiation.

The role of TG1 in bone remodeling in vivo is unknown. TG1, which is best known as ‘keratinocyte TG’, is mainly expressed by the stratified squamous epithelium. TG1 plays an essential role in keratinocytes where it crosslinks the cornified envelope proteins, which act as a protective skin barrier between the body and its environment [15,36]. Since the global knockout of *Tgm1* is lethal and pups die 4–5 h after birth due to dehydration, no skeletal phenotype has been reported for these mice [33,37]. Examining the skeletal phenotype of conditional TG1 knockout in osteoclasts will reveal whether it is a significant modulator of bone mass. This work is ongoing in our laboratory.

The role of FXIII-A in osteoclastogenesis is not entirely clear. It is abundant in BMMs but is downregulated immediately upon treatment of BMMs with RANKL so that mature osteoclasts do not express or have any FXIII-A activity. However, Raghu et al. [35] clearly demonstrated that *F13a1*−/− mice are protected from collagen-induced inflammatory arthritis and bone destruction which is due to defective osteoclast formation [35]. Thus, FXIII-A may have a role in some initial steps of osteoclastogenesis that is induced under inflammatory conditions. The increased osteoclastogenesis in *Tgm2*−/−; *F13a1*−/− double-knockout mice, however, shows that FXIII-A’s absence cannot block osteoclastogenesis in the absence of TG2 [19].

TG1 and FXIII-A are the only TG enzymes whose activity is promoted by proteolytic cleavage and the release of an activation peptide from the N-terminus of the enzymes. The activation of TG1 can occur via an aspartate protease, with Cathepsin D-mediated proteolytic cleavage resulting in the cornified envelope protein crosslinking during epidermal differentiation [33,38]. TG1 is initially synthesized as an inactive zymogen with a molecular weight of 106 kDa. It is expressed at low levels in proliferating keratinocytes and its expression is increased progressively during keratinocyte differentiation. TG1 is mostly bound to the plasma membrane and undergoes proteolytic cleavage, and forms a highly active complex of 100/67/33-kDa during the differentiation of keratinocytes [39]. In this paper, we show that the inhibition of the aspartate protease inhibitor Pepstatin A blocks osteoclastogenesis and inhibits TG1 activity in *Tgm2*−/− osteoclasts. We also show the fragmentation of TG1 in osteoclasts into molecular weight fragments similar to what is reported in skin and an increase in full-length TG1 upon Pepstatin A treatment, suggesting aspartate protease involvement in TG1 levels and activity. The observed high-molecular-weight form of TG1 is also seen, which could represent a similar complex of the activated fragments to that in keratinocytes. The exact size and identity of the fragments of TG1 remains unknown; however, because they are very close to the activated TG1-fragments reported in skin, they may harbor the increased TG activity seen in our analysis.

Our data also show a slight but significant increase in Cathepsin D activity in *Tgm2*−/− osteoclasts, making it a possible candidate aspartate protease that is involved in the activation. Several studies have shown the expression of aspartate protease in osteoclasts and their contribution to bone resorption (i.e., osteoclast activity) as they are active in a the low-pH microenvironment of resorption pits [32]. Results reported in the literature also indicate that Pepstatin A can suppress RANKL-induced multinuclear osteoclast formation through the inhibition of ERK phosphorylation and nuclear factor of activated T cells c1 (NFATc1) expression [40]. The possible role of other aspartic proteases in bone remodeling in vivo is not known. The aspartic protease family includes Cathepsin D, Cathepsin E, Renin, Pepsin, and Chymosin [41]. Our data show that bone marrow cells express the mRNA of at least Cathepsin D, Cathepsin E, and Renin. Whether Cathepsins or Cathepsin D can activate TG1 directly via cleavage in pre-osteoclasts or osteoclasts remains unknown and will be a goal of our future studies.

TG2 has been described as a Swiss army knife enzyme with numerous functions ranging from enzymatic to non-enzymatic activities [42]. TG2 plays a role in cell adhesion on the cell surface and ECM through a crosslinking function. In addition to protein crosslinking, several noncatalytic functions for TG2 have been reported [43]. Ca^2+^-ions and GTP (guanidine triphosphate) are important regulators of TG2. In the presence of GTP, TG2 has a compact/closed and inactive conformation, and in the presence of Ca^2+^-ions, it adopts an open conformation, making the catalytic core accessible to the substrates [44]. TG2 has also been reported to have five transcriptional variants or isoforms with unique functions and catalytic activities [45,46]. The regulation of Cathepsin D activity by TG2 was first described in mouse embryonic fibroblasts, where TG2 regulates the apoptosis level through the depletion of Cathepsin D by cross-linking the enzyme [34]. It is also documented that TG2 protects liver tissue from TNF-α dependent septic shock by reducing the level of Cathepsin D [47]. The molecular mechanisms of how TG2 can increase Cathepsin D activity involve crosslinking activity by TG2, i.e., TG2 can create Cathepsin D oligomers that have decreased activity. In our system, Cathepsin D oligomerization (Figure 7B), as a measure of non-active Cathepsin D, was not observed, and only the monomer form was detected with WB. An intriguing possibility is also that TG2 could regulate cellular pH via the release of ammonia as a product of isopeptide bond formation. The catalytic activity of aspartic proteases is optimal acidic pH [32,48]. The absence of the crosslinking process would decrease local ammonia levels, which would lower the pH, which, in turn, could increase aspartic protease activity. TG2 could also regulate Cathepsin D activity via deamination of the enzyme, which can occur in low-pH environments. These mechanisms should be investigated in situ in cells but demand sophisticated enzymatic activity probes and visualization methods. However, pH regulation may be a relevant factor as it is also well established that osteoclastogenesis is increased in a low-pH environment [49,50,51].

The mechanisms of how TG activity could ultimately promote osteoclastogenesis can only be speculated and may involve several pathways. The process of osteoclast differentiation and fusion is complex and involves a wide array of downstream signaling, resulting in the robust expression of NFATc1 (nuclear factor of activated T cell 1, which regulates RANKL-induced osteoclast fusion and osteoclast activation in a Ca^2+^-oscillation-dependent manner [52]. TGs, as Ca^2+^-dependent enzymes, may be an important part of the Ca^2+^ regulatory pathway. Interestingly, it was reported that TG1 forms of 67 and 33 kDa with higher TG activity increase in normal human foreskin epidermal keratinocytes upon increasing intracellular Ca^2+^ levels induced by ionophore A23187 [30]. A detailed analysis of TG1 regulation during osteoclastogenesis and proteomics analysis of all the substrate proteins via Hitomi peptide purification will allow for a better understanding of the pathways they are involved in.

In conclusion, our results suggest that the increased osteoclastogenesis in the absence of TG2 results from increased activity from TG1. This supports a novel role for TG1 in osteoclast differentiation. Also, importantly, our data show that TG2 can regulate the activity of other TGs. This is an area that should be taken into consideration when analyzing TG2 knockout phenotypes, which may also arise from the reminiscent or altered crosslinking activities of other TG family members.

## Data Availability

Not applicable.

## Supplementary Materials

The following supporting information can be downloaded at: https://www.mdpi.com/article/10.3390/cells12172139/s1, Figure S1: Validation of 5-(biotinamido)pentylamineand biotinylated Hitomi-peptides used for measuring TG activity; Figure S2: Increased formation of TRAP-positive (TRAP+) multinucleated cells in the absence of TH2; Figure S3: Increased osteoclast formation from *Tgm2*−/− bone marrow macrophages as visualize by actin ring formation; Figure S4: FXIII-A activity in Tgm2−/− bone marrow macrophages and osteoclasts; Figure S5: TG activities in *Tgm2*−/− bone marrow flushes; Figure S6: Evaluation of mRNA expression of TG1 and FXIII-A genes in bone marrow macrophages and osteoclasts.
