# Supplementary material for: Increased Osteoclastogenesis in Absence of TG2 Is Reversed by Transglutaminase Inhibition—Evidence for the Role for TG1 in Osteoclast Formation
Source: Cells. 2023 Aug 24;12(17):2139. doi: 10.3390/cells12172139 (PMC10487146; doi:10.3390/cells12172139)
Supplement: Supplementary file 1 [file cells-12-02139-s001.zip › cells-2460222-supplementary.pdf]

Supplemental Figure S1

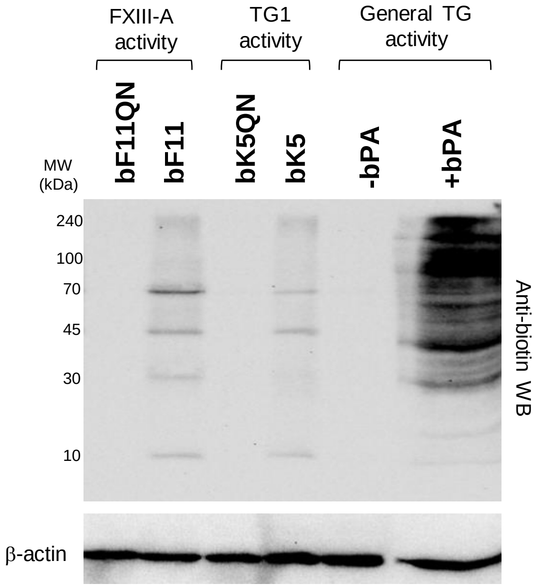

Supplemental Figure S1: Validation of 5-(biotinamido)pentylamine and biotinylated Hitomi-peptides used for measuring TG activity. The enzymatic activity of FXIIIA and TG1 were measured in the extracted protein from BMMs cells using specific Hitomi-substrate biotinylated peptides (bF11 and bK5, respectively) and their control peptides (bF11QN and bK5QN). Total TG activity was also assessed via primary amine, 5-(biotinamido)pentylamine (bPA). bPA was excluded in the negative control. The reaction mixtures were subjected to 10% SDS/PAGE followed by Western blotting and detection of biotin incorporation to substrate proteins in the extracts with anti-biotin antibody. No biotin incorporation to the substrate proteins was found in the control groups.

Supplemental Figure S2

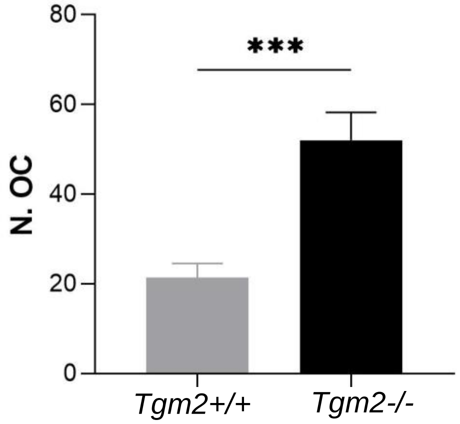

Supplemental Figure S2: Increased formation of TRAP-positive (TRAP+) multinucleated cells in the absence of TG2. BMMs cells from *Tgm2*-/- mice and control mice were treated with M-CSF and RANKL for 6 days followed by TRAP staining at end point. The number of TRAP+, multinucleated (≥3 nuclei) cells were calculated from microscope images. Osteoclast numbers in cultures are significantly higher in the absence of TG2. The data represent the mean of three separate experiments performed in triplicate; from where three separate image fields were used for counting the cell numbers. Bars represent mean of the quantification±SEM. n=9. *p<0.05.***p<0.001. ***p < 0.001.

Supplemental Figure S3

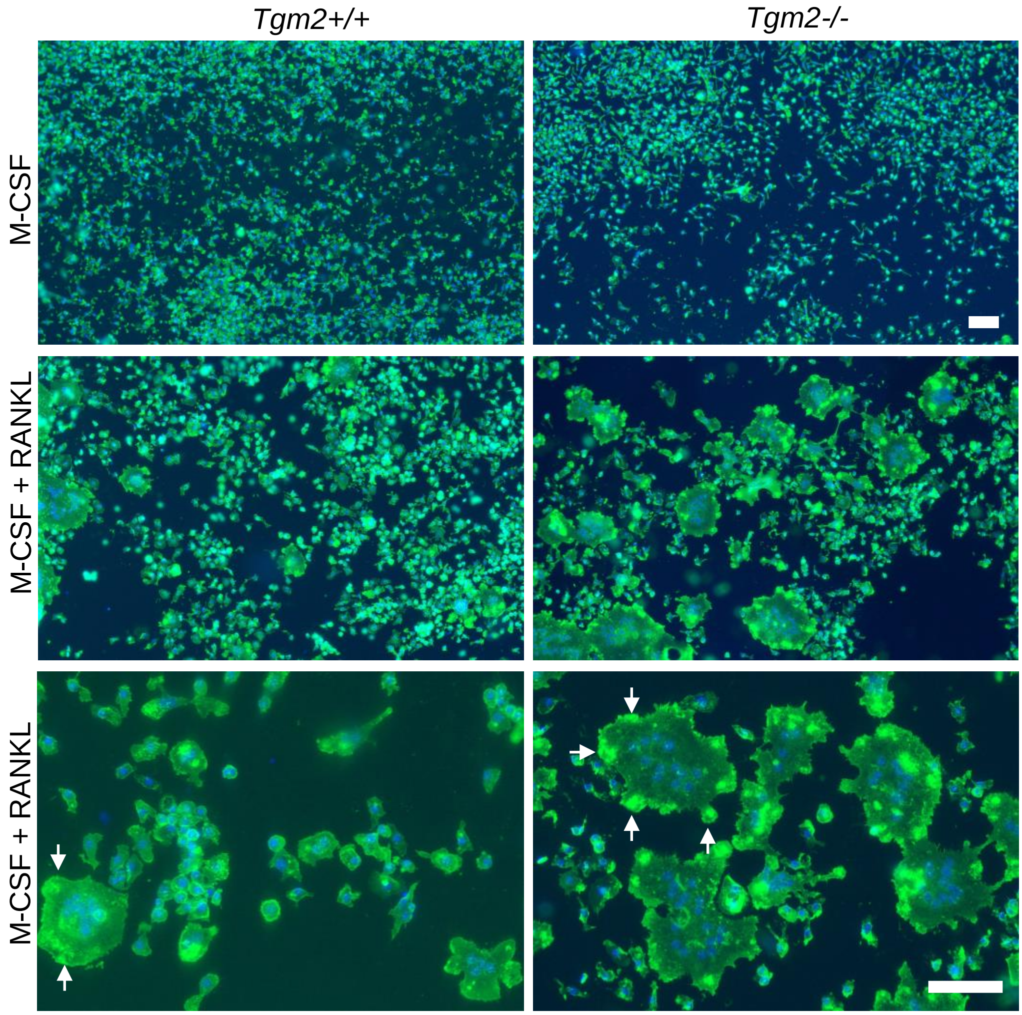

Supplemental Figure S3: Increased osteoclast formation from *Tgm2-/-* bone marrow macrophages as visualize by actin ring formation. BMMs cells from *Tgm2-/-* mice and control mice were cultured with M-CSF and RANKL for 6 days. Cells were then stained with Alexa Fluor® 488-phalloidin (green) and DAPI to visualize F-actin and nuclei, respectively. The formation of the large multinucleated osteoclasts with podosome structures (white arrows) is visibly increased in *Tgm2-/-* cultures compared to normal cells. Podosome structures are known to merge to form actin ring. Cells here are cultured on glass coverslips. Actin rings are often better visible on plastic surfaces. Lower panels are of higher magnification from different fields of M-CSF+RANKL treated cells. Magnification bar equals to 40 µm.

Supplemental Figure S4

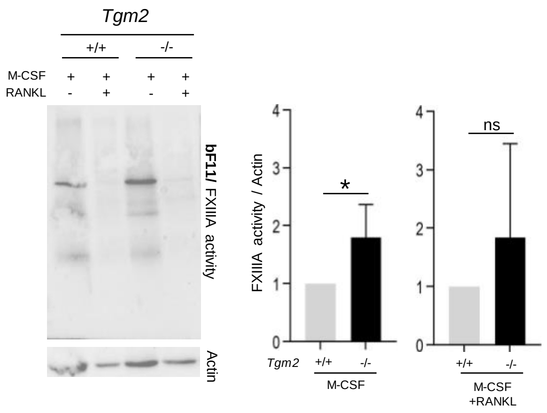

Supplemental Figure S4. FXIII-A activity in *Tgm2*-/- bone marrow macrophages and osteoclasts. Bone marrow macrophages (BMMs) isolated from *Tgm2*-/- and *Tgm2*+/+ mice were treated with M-CSF to maintain cells as BMMs or M-CSF+RANKL to induce osteoclastogenesis. At day 6 days cells were extracted and FXIII-A activity were assessed with bF11 peptide and Western blotting method. BMMs showed a a significant increase in FXIII-A activity in *Tgm2*-/- BMMs compared to WT cells, but no change in osteoclasts, which have a negligible level of FXIII-A activity. Western blot experiments normalized with respect to β-actin using NIH Image J. All statistical analyses were done with Student's T-test between knockout cells and WT cells (set to 1). Graphs do not reflect changes in activity between BMMs and osteoclasts. The data is expressed as the mean ± SEM, n=6. *p < 0.05; ns, non-significant.

Supplemental Figure S5

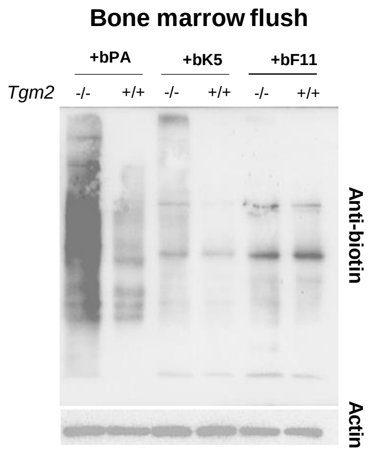

Supplemental Figure S5: TG activities in *Tgm2-/-* bone marrow flushes. Bone marrow flushes were prepared from *Tgm2*-/- and *Tgm2*+/+ mice by centrifugation of dissected femurs. Flushed were dissolved in lysis buffer, sonicated briefly after which protein concentration was measured. Total TG activity as well as TG1 and FXIII-A activities were measured using 5-(biotinamido)pentylamine and biotin-Hitomi-peptides, bK5, and bF11, respectively. Western blotting and detection with anti-biotin antibody shows the incorporation of bPA and bK5 and bF11 to substrates in flushes. Total activity and TG1 activity is visibly increased in knockout (*Tgm2*-/-) vs control (+/+) flushes. FXIIIA activity remains unchanged.

Supplemental Figure S6

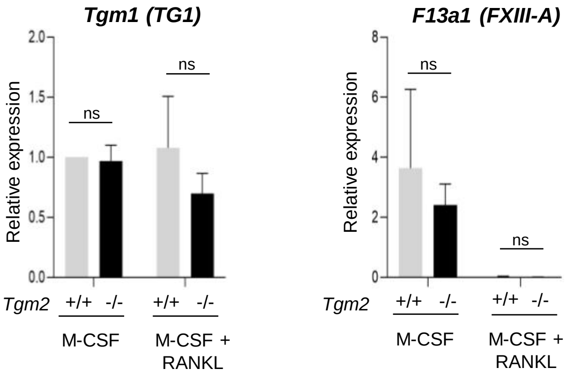

Supplemental Figure S6: Evaluation of mRNA expression of TG1 and FXIII-A genes in bone marrow macrophages and osteoclasts. Bone marrow macrophages (BMMs) of *Tgm2*-/- and control mice were treated with M-CSF and M-CSF+RANKL for 6 days to either maintain them as BMMs or induce osteoclastogenesis, respectively. mRNA was isolated and expression of *Tgm1* and *F13a1* genes was evaluated by qRT-PCR. *Tgm1* and *F13a1* mRNA expression showed no significant changes in *Tgm2*-/- cells compared to the wild type. *Tgm1* is expressed by both BMMs and osteoclasts, whereas *F13a1* is completely downregulated in osteoclasts. Data represents the mean of three separate experiments performed in triplicate ±SEM. n=9. *p<0.05.***p<0.001. ns; non-significant.
